# Cervical carcinoma with histologic components of sarcomatoid carcinoma and multiple basaloid variants: A case report and review of the literature

**DOI:** 10.1016/j.gore.2021.100797

**Published:** 2021-05-27

**Authors:** Paulina J. Haight, Antonio V. Castaneda, Johanna M. Savage, Larry J. Copeland

**Affiliations:** a Division of Gynecologic Oncology, The Ohio State University Wexner Medical Center, M210 Starling Loving Hall, 320 W 10^th^ Avenue, Columbus, OH 43210, United States; b Division of Pathology, The Ohio State University Wexner Medical Center, S305E Rhodes Hall, 410 W 10^th^ Avenue, Columbus, OH 43210, United States

**Keywords:** Basaloid squamous cell carcinoma, Sarcomatoid carcinoma

## Abstract

Cervical cancer with co-existing pathologic components of squamous cell carcinoma, basaloid morphology and sarcomatoid carcinoma is rare, with limited reports in the literature. Here we present a patient who underwent a modified radical hysterectomy for cervical cancer, with final pathology specimen demonstrating multiple histologic variants including basal carcinoma, adenoid cystic-like areas, basaloid squamous cell carcinoma and areas of high-grade transformation to sarcomatoid carcinoma.

## Highlights

• Basaloid cervical tumors are well-documented histologic variants and rarely exist in pure forms.
• Sarcomatoid carcinoma of the cervix is not yet classified as a histologic variant by the WHO.
• Squamous cell, basaloid and sarcomatoid carcinoma within a single cervical specimen is rare.

## 1 Introduction

Cervical cancer incidence in the United States is projected at 14,480 for the year 2021, with 4,290 estimated deaths (Siegel et al., 2021). While most cases of cervical cancer are squamous cell carcinomas, less common subtypes include adenocarcinoma, adenosquamous carcinoma, sarcoma, lymphoma, melanoma, and metastatic tumors. Additionally, the World Health Organization (WHO) Classification of gynecological tumors distinguishes several histological variants of cervical squamous cell carcinoma, including basaloid, among others (Pirog et al., 2019). Examples of basaloid histology can further be subdivided into basaloid squamous cell carcinoma, adenoid basal epithelioma, adenoid basal carcinoma, and adenoid cystic carcinoma. Basaloid tumors rarely exist as “pure” forms and are often admixed within a background of squamous cell carcinoma (Shi et al., 2020). Alternatively, sarcomatoid carcinoma of the cervix is a rarer histologic variant that is not listed within the WHO classification of gynecological tumors but has been described in few case reports and series (Lin et al., 2006, Brown et al., 2003, Hrudka et al., 2020, Mohan et al., 2008, Nageeti and Jastania, 2012). According to these reports, it an aggressive tumor with relatively poor clinical outcome.

Here, we present the rare phenomenon of a single cervical tumor specimen of invasive carcinoma with both basaloid histologic components in addition to high grade sarcomatous transformation.

## 2 Case report

**Fig. 1:**
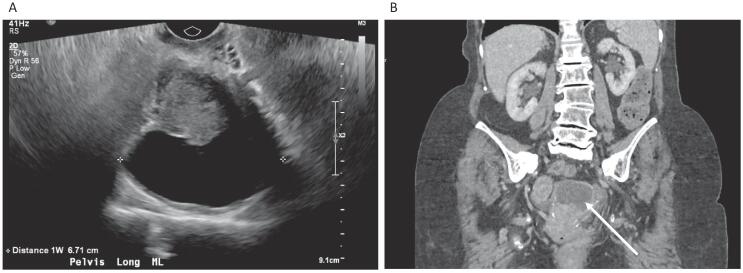
Pre-operative imaging. Legend. (A) Pelvic ultrasound demonstrating transverse image of the uterus, with cystic lesion at the uterine fundus measuring over 6 cm in greatest dimension, (B) CT abdomen/pelvis demonstrating pelvic cystic lesion (arrow).

A 91-year-old initially presented with several months of brown vaginal discharge following a conservatively managed pelvic fracture. A CT of the hip and pelvis confirmed a non-displaced fracture of the left pubic ramus in addition to an indeterminant 5.4 cm fluid collection in the pelvic cul-de-sac, questionably arising from the uterus or ovary. The cystic lesion was persistent on a CT scan of the abdomen and pelvis 4 months later (Fig. 1), without any evidence of retroperitoneal lymphadenopathy or metastatic disease. A pap test resulted as ASCUS, cannot rule out ASC-H. There were no cervical lesions noted on colposcopy, and an endocervical curettage pathology showed atrophic squamous epithelium without dysplasia. A pelvic ultrasound showed an indeterminate endometrial stripe, possible endometrial and cervical calcifications, and a slight increase in size of the cystic pelvic lesion at 6 cm, which was located superior to the uterus, as seen in Fig. 1. CA-125 was normal. Hysteroscopy, dilation and curettage was performed with intra-operative findings consistent with atrophic cervical and endometrial epithelium, but no mass or abnormalities otherwise. Surgical pathology of the endometrial curettage resulted as poorly differentiated squamous cell carcinoma, which was HPV DNA in situ hybridization positive (family 16), supporting cervical origin.

**Fig. 2:**
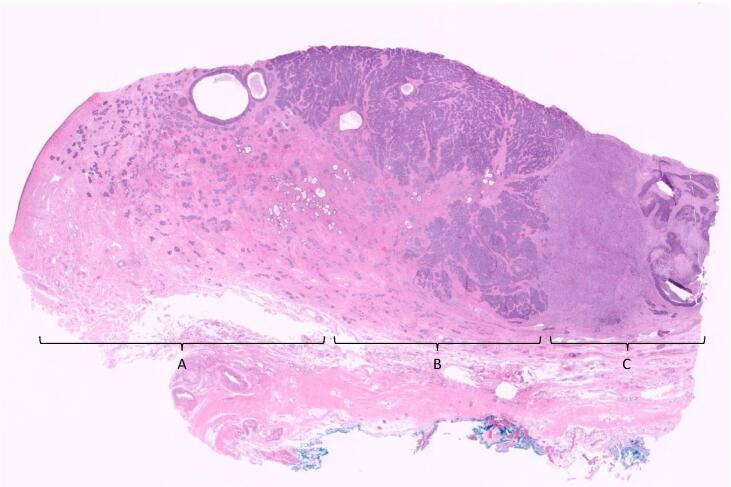
Invasive cervical carcinoma with multiple histologic variants. Legend. Deeply invasive cervical carcinoma with admixed basaloid morphology and high-grade/sarcomatous transformation (H&E, 20x total magnification). Region A corresponds to areas of adenoid basal epithelioma and adenoid basal carcinoma; Region B demonstrates transformation to adenoid cystic-like and basaloid squamous cell carcinoma; Region C corresponds to an area of sarcomatoid transformation.

**Fig. 3:**
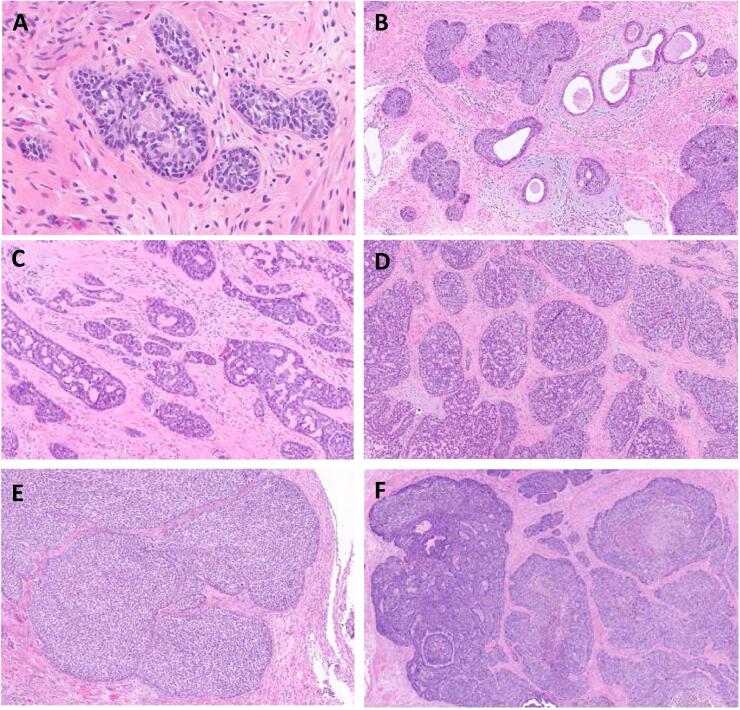
Spectrum of basaloid morphology within the tumor. Legend. (A-B) Areas of the neoplasm consistent with adenoid basal epithelioma and adenoid basal carcinoma (H&E, 400x and 100x total magnification), (C-D) Areas of the neoplasm with adenoid cystic-like features (H&E, 100x total magnification), (E-F) Areas of the neoplasm consistent with basaloid squamous cell carcinoma (H&E, 100x and 50x total magnification).

**Fig. 4:**
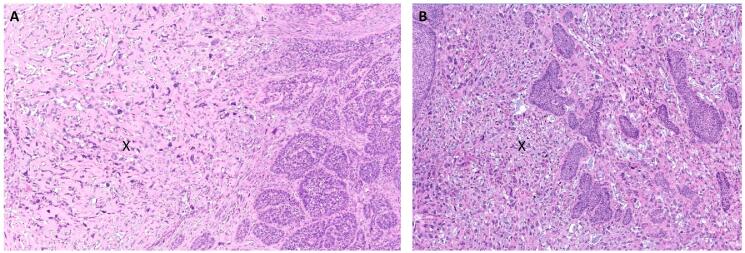
Sarcomatoid carcinoma within the tumor. Legend. High-grade transformation to sarcomatoid carcinoma. The sarcomatoid component is designated by an “X” in images A and B (H&E, 200x total magnification).

The patient was seen by gynecologic oncology and noted to have a normal in-office examination. She was recommended to undergo exploratory laparotomy, radical hysterectomy, bilateral salpingo-oophorectomy and pelvic lymph node sampling for suspected early-stage cervical cancer based on interdisciplinary review of her prior surgical pathology specimen, normal physical exam and absence of obvious metastatic disease on prior imaging. Intra-operative findings were significant for nodularity of the anterior cervix. There was no extra-cervical disease, lymphadenopathy or evidence of distant metastatic disease. Final pathology revealed deeply invasive cervical carcinoma with adenoid basal/basaloid/squamous/adenoid-cystic-like differentiation and high-grade transformation consistent with sarcomatoid carcinoma, as seen in Fig. 2, Fig. 3, Fig. 4. The tumor specimen was 5.5 cm in greatest dimension, extensively involving the cervix and parametria. Deep stromal invasion was noted however could not be verified in millimeters as the tumor invaded the cervical wall and involved the anterior and posterior paracervical soft tissue margins. Lymphovascular invasion was present. Two lymph nodes were sampled (one left pelvic, one right pelvic), and nodal status was negative. Pathologic evaluation was consistent with FIGO stage IIB disease. The case was presented for review at our institution’s tumor board, where the recommendation was made for pelvic external beam radiation therapy. Concurrent chemotherapy was foregone due to patient age. The patient underwent 21 out of 25 fractions (total dose 3780 cGy) of external beam radiation therapy prior to discontinuation due to side effects. She is currently doing well with no evidence of disease at 5 months of follow-up.

## 3 Discussion

Our patient’s surgical specimen demonstrated multiple histologic variants (Fig. 2). The range of basaloid histologies included: basaloid squamous cell carcinoma, adenoid basal epithelioma, adenoid basal carcinoma and adenoid cystic-like areas (Fig. 3). There were also regions of the tumor that demonstrated high-grade transformation with marked pleomorphism and spindle-cell change. These stained strongly positive for cytokeratins, arguing against a biphasic tumor and towards a sarcomatoid carcinoma (Fig. 4). To our knowledge, the individual histologic components of our patient’s neoplasm are rare, and the combination is even more unique.

Although uncommon, it is well-documented that squamous cell carcinomas of the cervix may demonstrate basaloid histology and are generally called “basaloid squamous cell carcinomas.” Additional basaloid histologic variants include adenoid basal epithelioma, adenoid basal carcinoma and adenoid cystic carcinoma. These variants exist on a spectrum of low-grade lesions to very aggressive tumors. Adenoid basal epithelioma describes a histologically low-grade lesion that follows a benign clinical course. Adenoid basal carcinoma has more infiltrative architectural features with cytologic change and is often seen with a background of adenoid basal epithelioma. Adenoid cystic carcinoma is rare and is associated with poor prognosis due to frequent local recurrence and distant metastases. Basaloid tumors rarely exist as “pure” forms and are often admixed with other basaloid histologic variants in addition to squamous cell carcinoma. In less than 1% of all cervical carcinomas, a pure adenoid basal carcinoma or adenoid cystic carcinoma has been described (Shi et al., 2020). Given that a majority of these neoplasms demonstrate histological components of squamous cell carcinoma, they are often treated as such.

Sarcomatoid carcinoma has not been recognized as a histologic variant of cervical cancer per the WHO Classification of gynecological tumors, but it has been described in several case reports and series (Brown et al., 2003, Hrudka et al., 2020, Mohan et al., 2008, Nageeti and Jastania, 2012). More robust literature exists describing sarcomatoid carcinomas of the head and neck, which denote a poor prognosis from conventional squamous cell carcinoma despite aggressive surgical and adjuvant therapies (Chang et al., 2013). Sarcomatoid carcinoma is an epithelial tumor that mimics features of a sarcoma, but no malignant mesenchymal component. It is histologically comprised of a heterogenous tumor cell population; areas consistent with typical squamous cell carcinoma can be seen, in conjunction with spindle-cell morphology characteristic of a sarcoma. Bizarre, multinucleated giant cells have also been documented. Immunohistochemical analysis will show spindle-cell positivity for cytokeratins, indicating epithelial origin. The mesenchymal marker Vimentin is also typically expressed (Brown et al., 2003). There is inconsistent data as to whether sarcomatoid carcinoma of the cervix is associated with HPV. HPV16 and HPV33 have been reported in prior cases, in addition to negative HPV staining (Hrudka et al., 2020, Mohan et al., 2008, Lin et al., 2006). Our patient was positive for HPV16 via in situ hybridization, although this could be due to the additional basaloid morphologic variants which frequently demonstrate HPV16 and HPV18 positivity (Shi et al., 2020, Missaoui et al., 2018).

Sarcomatoid carcinoma of the cervix, despite limited literature, is described as an aggressive tumor with poor clinical outcome, much like its counterpart in other organs, including head and neck (Chang et al., 2013). Patients often present with advanced disease but for those with early-stage disease, primary treatment offers the best chance of cure. An argument has been made for pelvic radiation being the most effective treatment modality (Brown et al., 2003, Mohan et al., 2008). In the largest case series published by Brown et al, all 9 patients had complete response to initial therapy, however more than half recurred within a short disease-free interval (median 4.9 months). At the time of recurrence, most patients are noted to have widespread metastases and succumb to the disease shortly thereafter (Brown et al., 2003).

It is important to take into account the most aggressive histologic subtypes of a tumor when considering treatment and prognosis. For our patient, these would be the adenoid cystic carcinoma and sarcomatoid carcinoma components. She underwent adjuvant pelvic radiation therapy, which seems to be the most effective treatment modality according to our literature review. In a younger patient we might also advocate for the addition of chemotherapy in order to provide radiosensitizing benefits in combination with radiotherapy. This more aggressive treatment regimen in the primary setting may be warranted given these tumors’ propensity for early disease progression and failure of subsequent treatment options. However, chemotherapy was foregone in our particular case due to the patient‘s age and medical co-morbidities.

## 4 Conclusion

Cervical cancers may demonstrate a variety of histological components, with some being rarer. Here we describe a report of multiple basaloid morphologies in addition to sarcomatoid carcinoma diagnosed within the same pathologic specimen.

## Informed consent

Informed consent was obtained from the patient for publication of this case report. A copy of the written consent is available for review by the Editor-in-Chief of this journal on request.

## CRediT authorship contribution statement

**Paulina J. Haight:** Conceptualization, Data curation, Formal analysis, Investigation, Writing - original draft. **Antonio V. Castaneda:** Conceptualization, Data curation, Formal analysis, Investigation, Writing - review & editing. **Johanna M. Savage:** Data curation, Visualization, Writing - review & editing. **Larry J. Copeland:** Conceptualization, Formal analysis, Supervision, Writing - review & editing.

## Declaration of Competing Interest

The authors declare that there is no conflict of interest.
